# Myxobacterial Response to Methyljasmonate Exposure Indicates Contribution to Plant Recruitment of Micropredators

**DOI:** 10.3389/fmicb.2020.00034

**Published:** 2020-01-28

**Authors:** Barbara I. Adaikpoh, Shukria Akbar, Hanan Albataineh, Sandeep K. Misra, Joshua S. Sharp, D. Cole Stevens

**Affiliations:** Department of BioMolecular Sciences, The University of Mississippi, Oxford, MS, United States

**Keywords:** myxobacteria, methyljasmonate, rhizobiome, micropredator, phytohormones

## Abstract

Chemical exchanges between plants and microbes within rhizobiomes are critical to the development of community structure. Volatile root exudates such as the phytohormone methyljasmonate (MeJA) contribute to various plant stress responses and have been implicated to play a role in the maintenance of microbial communities. Myxobacteria are competent predators of plant pathogens and are generally considered beneficial to rhizobiomes. While plant recruitment of myxobacteria to stave off pathogens has been suggested, no involved chemical signaling processes are known. Herein we expose predatory myxobacteria to MeJA and employ untargeted mass spectrometry, motility assays, and RNA sequencing to monitor changes in features associated with predation such as specialized metabolism, swarm expansion, and production of lytic enzymes. From a panel of four myxobacteria, we observe the most robust metabolic response from plant-associated *Archangium* sp. strain Cb G35 with 10 μM MeJA impacting the production of at least 300 metabolites and inducing a ≥ fourfold change in transcription for 56 genes. We also observe that MeJA induces *A.* sp. motility supporting plant recruitment of a subset of the investigated micropredators. Provided the varying responses to MeJA exposure, our observations indicate that MeJA contributes to the recruitment of select predatory myxobacteria suggesting further efforts are required to explore the microbial impact of plant exudates associated with biotic stress.

## Introduction

Complex communities of microbes within rhizobiomes significantly benefit plant health (Berendsen et al., 2012; Bulgarelli et al., 2013; Muller et al., 2016; Richter-Heitmann et al., 2016; Sasse et al., 2018; Olanrewaju et al., 2019; Poudel et al., 2019). The role that plants might play in curating these microbial populations remains mostly theoretical (Lundberg et al., 2012; Bulgarelli et al., 2013; Chaparro et al., 2014; Schreiter et al., 2014; Reinhold-Hurek et al., 2015; Muller et al., 2016; Zgadzaj et al., 2016; Berendsen et al., 2018; Sasse et al., 2018). However, metagenomic studies have indicated numerous plant species maintain distinct rhizobiomes (Bulgarelli et al., 2012; Peiffer et al., 2013; Schlaeppi et al., 2014; Shi et al., 2015; Coleman-Derr et al., 2016; Kawasaki et al., 2016; Zgadzaj et al., 2016; Sasse et al., 2018). While a variety of factors contribute to these organized communities, volatile exudates or volatile organic compounds such as the phytohormone methyljasmonate (MeJA) are associated with defense responses utilized by plants to combat pathogenic microorganisms and herbivorous insects (Junker and Tholl, 2013; Carvalhais et al., 2015; Lugtenberg et al., 2017; Massalha et al., 2017; Sasse et al., 2018; Liu and Brettell, 2019). Considering the requirement of these exudates to be produced at titers sufficient for communication throughout soils, exudates might also facilitate recruitment or maintenance of beneficial microbial populations of the root microbiome (Sasse et al., 2018). Microbes belonging to one such group of bacteria considered beneficial, the Myxococcales, more colloquially referred to as myxobacteria, are micropredators that are ubiquitous in soils and capably predate microbes from genera that include plant pathogens (Dawid, 2000; Lueders et al., 2006; Perez et al., 2014; Zhou et al., 2014; Li et al., 2017; Livingstone et al., 2017; Cui et al., 2018; Petters et al., 2018; Lin et al., 2019). Predatory myxobacteria demonstrate complicated social features, contribute to carbon turnover, produce a variety of antimicrobial specialized metabolites, and significantly impact the microbial food web within soils (Bretl and Kirby, 2016; Findlay, 2016; Landwehr et al., 2016; Munoz-Dorado et al., 2016; Herrmann et al., 2017; Schumacher and Sogaard-Andersen, 2017; Mohr, 2018; Petters et al., 2018). Myxobacteria have also been observed to activate predatory features when exposed to exogenous, quorum signaling molecules typically produced by prey bacteria; this precedent for community signal perception and response makes myxobacteria excellent candidates for MeJA exposure experiments (Lloyd and Whitworth, 2017). Given their potential as pathogen-suppressing bacteria beneficial to root microbiomes, we sought to investigate how myxobacteria respond when exposed to ecologically equivalent titers of the phytohormone MeJA (Mueller et al., 1993; Blechert et al., 1995; Vijayan et al., 1998).

We suggest the following responses to MeJA exposure would support plant recruitment of myxobacteria: (1) global changes in metabolism, (2) increased motility to support recruitment, and (3) impacted production of predation-associated lytic enzymes. Myxobacteria are broadly referenced as beneficial to rhizobiomes without distinction between phylogenetically dissimilar members. For these experiments, the myxobacteria *Archangium* sp. strain Cb G35, *Corallococcus coralloides* strain M2, *Cystobacter ferrugineus* strain Cbfe23, and *Nannocystis pusilla* strain Na p2 were exposed to MeJA and induced metabolic responses were determined using untargeted mass spectrometry-based profiling (Zander et al., 2011; Adaikpoh et al., 2017; Livingstone et al., 2018b). The MeJA-impacted motilities of *A.* sp., *C. coralloides*, and *C. ferrugineus* and transcriptomic responses from the plant-associated myxobacterium *A.* sp. strain Cb G35, originally isolated from tree bark in India, were also determined using swarming assays and RNA sequencing (Zander et al., 2011; Adaikpoh et al., 2017).

## Results

### MeJA Exposure Impacts Myxobacterial Metabolism

**FIGURE 1:**
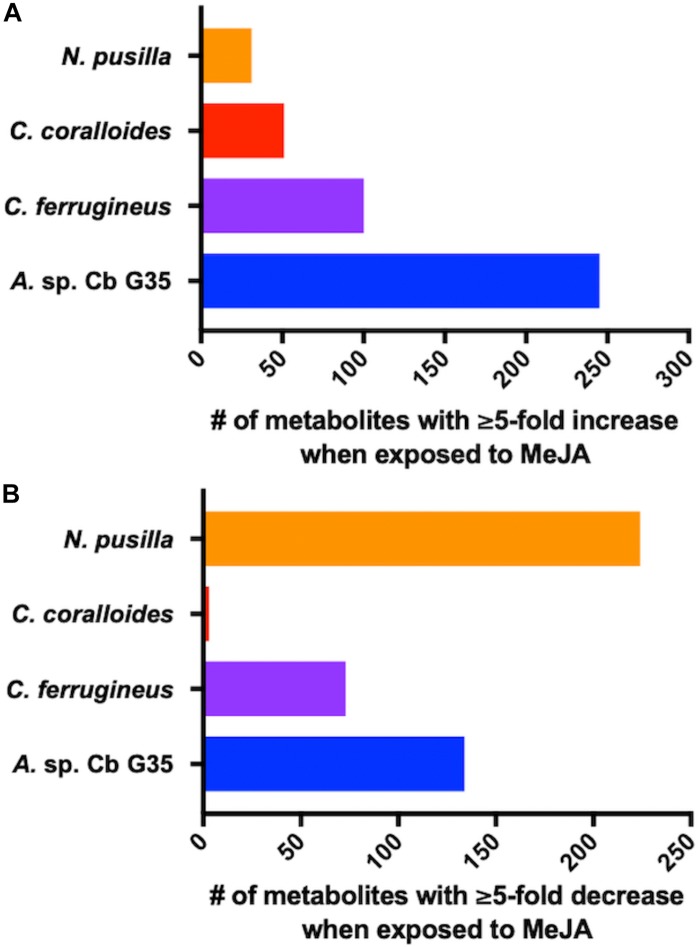
MeJA-impacted features provided by XCMS analysis of LC-MS/MS data after filtering feature tables for those with a ≥ fivefold change and *p* ≤ 0.05 with **(A)** depicting increased metabolites and **(B)** depicting decreased metabolites.

**FIGURE 2:**
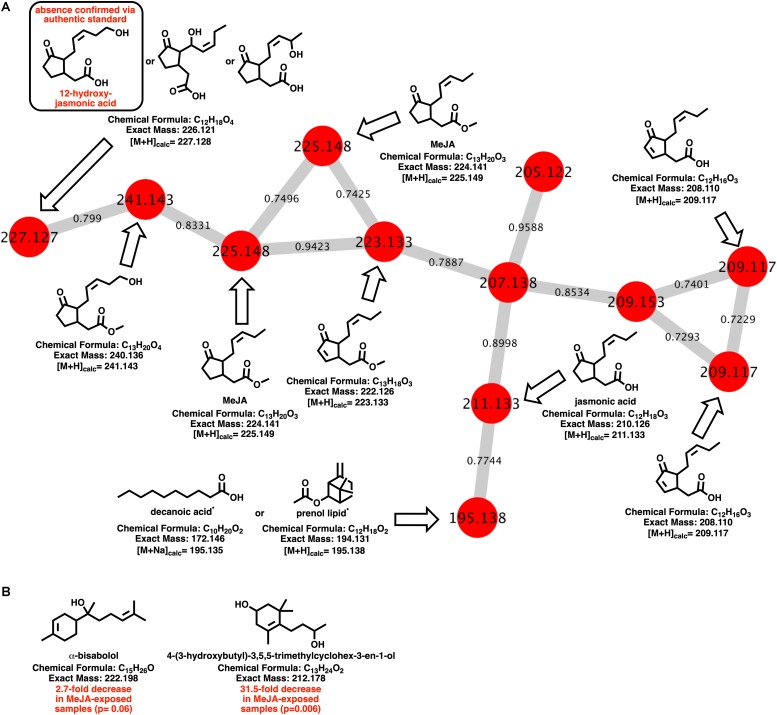
**(A)** Molecular network of MeJA and associated metabolites including nodes labeled with detected parent ions and edges labeled with cosine values. Predicted metabolites provided by NAP analysis of the GNPS-rendered cluster. *Multiple fatty acids and prenol lipids with identical exact masses predicted by NAP analysis. **(B)** Metabolites identified by GNPS analysis (cosine > 0.8) impacted by MeJA exposure.

Myxobacteria utilize a combination of antimicrobial specialized metabolites and lytic enzymes to facilitate consumption of prey (Findlay, 2016; Herrmann et al., 2017). If the plant phytohormone MeJA is involved in recruitment of myxobacteria to stave off pathogens, a shift in metabolism to produce predation-associated metabolites during exposure experiments might be observable. Utilizing XCMS-MRM to compare untargeted mass spectrometry datasets from MeJA exposed extracts against unexposed extracts, each investigated myxobacteria demonstrated a significant, albeit varied, metabolic shift (Figure 1) (Domingo-Almenara et al., 2018; Forsberg et al., 2018). From these datasets, we observe various changes in detected intensities ranging from 54–349 total metabolites with *C. coralloides* extracts being the least impacted and *A.* sp. being the most (Figure 1). Extracts from *A.* sp. had the most features with a ≥ fivefold increase in detection with a total of 245, and *N. pusilla* had the most features with a ≥ fivefold decrease in detection with a total of 224. Subsequent molecular networking of the datasets using the Global Natural Products Social Molecular Networking (GNPS) platform, suggested each myxobacteria capably metabolized MeJA to afford observable oxidized analogs (Wang et al., 2016). LC-MS/MS data from *A.* sp. extracts are available in a MassIVE Public GNPS dataset (MSV000083921). Considering the plant pathogen *Magnaporthe oryzae* oxidizes jasmonic acid to 12-hydroxy-jasmonic acid (colloquially referred to as tuberonic acid) to subvert jasmonate-associated immune response, we utilized the GNPS-affiliated, theoretical/*in silico* tool, Network Annotation Propagation (NAP) to explore the identity of the putative MeJA-associated metabolites (Wang et al., 2016; Patkar and Naqvi, 2017; da Silva et al., 2018). Structural predictions provided by NAP suggested all four investigated myxobacteria might produce any one of the following known jasmonic acid analogs 12-hydroxy-jasmonic acid, 11-hydroxy-jasmonic acid, or 8-hydroxy-jasmonic acid (227.127 *m/z*) as well as the known metabolite 4,5-didehydrojasmonate (223.133 *m/z*) previously isolated from *Jasminum grandiflorum* (Figure 2A) (Kaiser and Lamparsky, 1974; Kiyota et al., 1997). However, dissimilar retention times and fragmentation patterns of the oxidized MeJA metabolites when compared to a purchased standard confirmed that none of the investigated myxobacteria produce the pathogen-associated analog 12-hydroxy-jasmonic acid.

Of the features within *A.* sp. extracts identified by GNPS with high fragmentation similarities (cosine > 0.8) to databases entries, a total of two demonstrated a significant change in detected quantities when exposed to MeJA. The identified metabolites included α-bisabolol (205.195 *m/z*; [M + H-H_2_O]) and 4-(3-hydroxybutyl)-3,5,5-trimethylcyclohex-3-en-1-ol (213.185 *m/z*; [M + H]). Detected quantities of both metabolites were decreased in *A.* sp. extracts exposed to MeJA (Figure 2B). While *A.* sp. demonstrated the most robust metabolic response to MeJA exposure, the previously reported antimicrobial metabolite roimatacene was conspicuously absent from all generated extracts (Zander et al., 2011). However, this absence can reasonably be attributed to the difference in *A.* sp. cultivation conditions and medias combined with the reported instability of roimatacene (Zander et al., 2011).

### MeJA Exposure Influences *A.* sp. Motility

**FIGURE 3:**
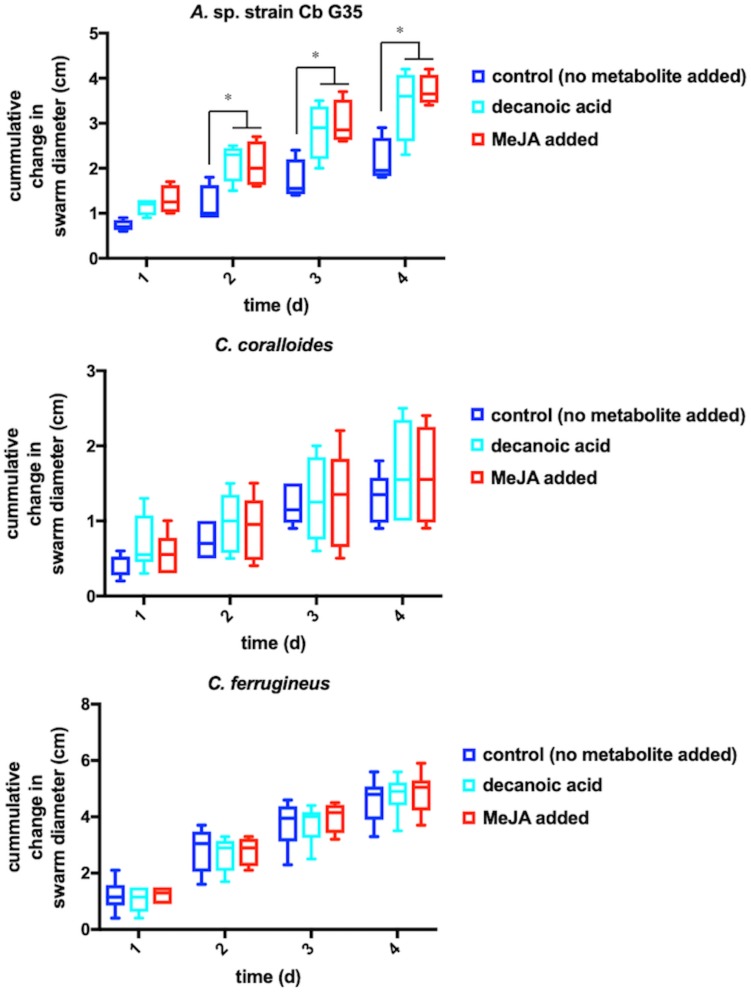
Motility assays depicting cumulative change in swarm diameter post MeJA exposure. The symbol “^∗^” indicates *P* < 0.05.

Any increased motility induced by MeJA exposure would also support plant recruitment of myxobacteria. Growth of myxobacteria in cooperative swarms affords the ability to observe growth as a function of swarm expansion rate (McBride et al., 1992; Zhou and Nan, 2017). The impact of MeJA exposure on swarm expansion rates was observed by measuring swarm diameters daily for MeJA exposed and unexposed myxobacteria. *N. pusilla* was excluded from motility assays due to the agarolytic growth and atypical swarming patterns. Each myxobacterium was also exposed to 10 μM decanoic acid due its previously reported impact on myxobacterial motility (McBride et al., 1992). A significant increase in *A.* sp. swarm diameters was observed in MeJA-exposed samples when compared to unexposed samples after 4 days of growth with the most significant increase in swarm diameter occurring after the first day of exposure (Figure 3). Interestingly, both decanoic acid and MeJA induced significant changes in *A.* sp. swarming. Of the three myxobacteria subjected to motility assays, only *A.* sp. demonstrated a response (Figure 3). Decanoic acid induced activation of the frizzy (*frz*) signal transduction pathway associated with chemotaxis has been reported from in the model myxobacterium *Myxococcus xanthus* (McBride et al., 1992). The similar responses between decanoic acid and MeJA exposures combined with the observed MeJA-induced metabolite from *A.* sp. predicted to be decanoic acid (Figure 2) suggests overlap between metabolic and motility responses.

### MeJA Exposure Induces Changes in *A.* sp. Transcriptome and Activates Transcription of Lytic Enzymes

**FIGURE 4:**
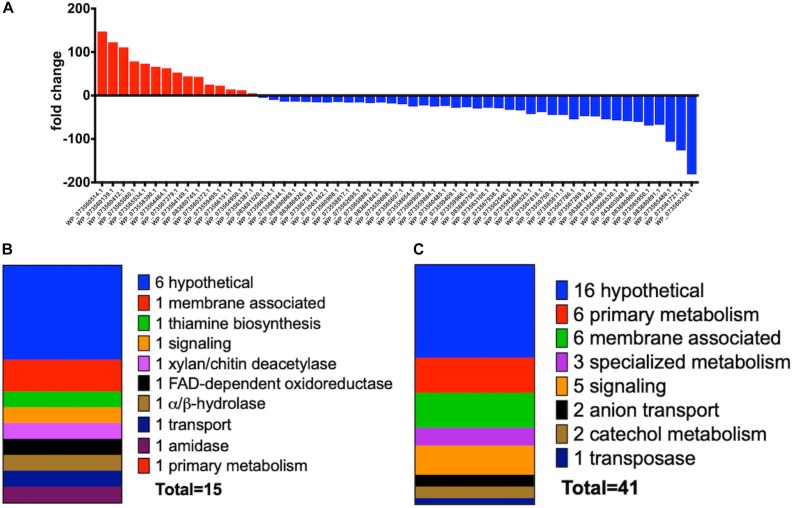
**(A)** Fold change data observed from *A.* sp. MeJA exposure experiments with increased transcription indicated with red and positive fold change values and decreased transcription indicated with blue and negative fold change values. Data represented for all genes with ≥ fourfold change (*P* < 0.05; *n* = 3 per condition). **(B)** General categories and classes associated with *A.* sp. genes with observed ≥ fourfold increases in transcription during MeJA exposure. **(C)** General categories associated with *A.* sp. genes with observed ≥ fourfold decreases in transcription during MeJA exposure.

Increased transcription of lytic enzymes induced by MeJA exposure would support exudate-associated predation and correlate with previously observed MeJA-induced metabolic shifts. Provided the robust metabolic response and MeJA impacting motility from *A.* sp., RNA sequencing was employed to provide transcriptomes for MeJA exposed and unexposed cultures of *A.* sp. Using the sequenced genome for *A.* sp. strain Cb G35 (NZ_MPOI00000000.1) as a reference, a total of 56 genes were determined to experience a statistically significant, > fourfold difference in expression (Tables 1, 2 and Figure 4) (Adaikpoh et al., 2017). A total of 15 overexpressed genes during MeJA exposure increased four to 147-fold including a 73-fold change for a predicted lytic amidase with an identified CHAP domain (Bateman and Rawlings, 2003; Becker et al., 2009). Other genes demonstrating increased transcription are associated with proteins that include a histidine kinase, a bifunctional hydroxymethyl pyrimidine kinase homologous to ThiD from thiamine biosynthesis, an ATP-binding ABC exporter with homology to CcmA, a radical *S-*adenosylmethionine (SAM) pyruvate-formate lyase activating enzyme, an α/β-hydrolase with homology to MhpC, and a NodB-like xylan/chitin deacetylase (Table 1) (John et al., 1993; Begley et al., 1999; Schulz et al., 1999; Leonardi and Roach, 2004; Dunn et al., 2005; Shisler and Broderick, 2014). A total of 41 genes were observed to be downregulated between four and 182-fold under MeJA exposure conditions (Table 2 and Figure 4). Interestingly, none of the upregulated genes induced by MeJA exposure obviously correspond to the observed shifts in metabolism, and genes impacted by MeJA with predicted roles in specialized metabolism were downregulated upon exposure.

**TABLE 1:** Associated gene products and fold change for genes with increased transcription during MeJA exposure conditions*^a^*.

Gene product	Description	Fold change
WP_073560412.1	FAD-dependent oxidoreductase	111
WP_073565080.1	DUF2085 containing membrane protein	79
WP_143195895.1	CHAP domain amidase	73
WP_073564464.1	Bifunctional hydroxymethyl pyrimidine kinase	63
WP_073560372.1	ABC transporter	25
WP_073559495.1	α/β hydrolase	23
WP_073566151.1	Radical SAM pyruvate-formate lyase activating enzyme	14
WP_143195831.1	Xylan/chitin deacetylase	12
WP_073563387.1	Histidine kinase	5

**^*a*^*Gene products annotated as hypothetical proteins omitted.*

**TABLE 2:** Associated gene products and fold change for genes with decreased transcription during MeJA exposure conditions*^a^*.

Gene product	Description	Fold change
WP_083681520.1	TOMM kinase cyclase	5
WP_073566534.1	Serine protease	10
WP_143195956.1	PAP2 family protein	14
WP_143195341.1	DUF 2254 domain-containing protein	14
WP_083680826.1	Membrane chloride channel	15
WP_073560908.1	Histidine kinase	15
WP_143195863.1	Sulfate transporter	16
WP_073567587.1	FAD-dependent oxidoreductase	16
WP_073562695.1	Chain-length determining protein	16
WP_083681843.1	IS21 family transposase	16
WP_073565888.1	Galactose oxidase	18
WP_073565697.1	Tyrosinase	20
WP_073560909.1	Protocatechuate 3,4-dioxygenase	23
WP_073558654.1	Glutathione-dependent formaldehyde dehydrogenase	25
WP_073559864.1	SAM-dependent methyltransferase	26
WP_073559966.1	Cholesterol acyltransferase	27
WP_073559409.1	DUF3616 domain-containing protein	28
WP_083680758.1	Cyclopropane fatty-acyl-phospholipid synthase	30
WP_073562546.1	ATP-dependent endonulease	33
WP_073558548.1	Cytochrome P450	34
WP_073566525.1	Osmotic shock protein	43
WP_073567786.1	Uma2 family endonuclease	55
WP_083680900.1	Histidine kinase-like ATPase	61
WP_073565950.1	Acyl-CoA thioesterase	69
WP_083680891.1	GntR family transcriptional regulator	67

**^*a*^*Gene products annotated as hypothetical proteins omitted.*

## Discussion

Analysis of extracts from four soil-associated myxobacteria using untargeted mass spectrometry demonstrated the varying metabolic shifts experienced by each myxobacterium upon exposure to MeJA. Our original assertion that this metabolic shift might be attributed to increased production of predation-associated specialized metabolites is not necessarily supported by our subsequent transcriptomic analysis of MeJA exposed *A.* sp. samples. This is perhaps best explained by the observation that myxobacteria constitutively produce predation-associated features and instead regulate subsequent feeding when exposed to lysed prey (Livingstone et al., 2018a). We suspect that continued exploration of this MeJA-impacted metabolic space utilizing the nutrient rich medias and increased cultivation volumes previously attributed to *A.* sp. antimicrobial production will facilitate natural product discovery and provide insight into this discrepancy (Zander et al., 2011). Interestingly, all of the library hits provided by GNPS analysis were mono- or sesquiterpene metabolites that have previously been considered plant metabolites (Schneider et al., 2001; Zhang et al., 2018; Muangphrom et al., 2019). Although production of α-bisabolol and characterization of the corresponding synthase (AB621339.1) from *Streptomyces citricolor* have been reported, no homology to this synthase was observed when comparing the six putative terpene synthases from *A.* sp. identified by antiSMASH (Nakano et al., 2011; Blin et al., 2019). While the previously referenced constitutive toxicity observed from myxobacteria and our observation of MeJA-impacted production of biologically active terpene metabolites is certainly intriguing, subsequent efforts are required for confirmation and characterization of the associated biosynthetic gene clusters.

This analysis also provided insight into myxobacterial metabolism of MeJA, and a variety of known jasmonate-associated metabolites were predicted using the GNPS-affiliated NAP platform (Wang et al., 2016; da Silva et al., 2018). The 111-fold increase in transcription of a predicted FAD-dependent oxidoreductase during MeJA exposure provides a likely candidate involved in the oxidation of MeJA to provide either of the oxidized MeJA analogs predicted to be either 8-hydroxy-jasmonic acid or 10-hydroxy-jasmonic acid and determined not to be the pathogen-associated metabolite 12-hydroxy-jasmonic acid (Patkar and Naqvi, 2017).

The MeJA-dependent presence of a metabolite predicted to be either a saturated fatty acid, including decanoic acid, or a prenol lipid (Figure 2A) is also intriguing as a variety of saturated fatty acids activate the frizzy (*frz*) signal transduction pathway associated with chemotaxis in the myxobacterium *M. xanthus* (McBride et al., 1992). This result also provides insight into the increased motility observed when exposing *A.* sp. to MeJA. While the transcription of the FrzCD homolog from *A.* sp., WP_073562522.1, was not impacted by MeJA exposure (Supplementary Material), FrzCD is a methyl-accepting chemotaxis protein (MCP) with elicitor-induced activation attributed to covalent modification via demethylation and not transcriptional activation (McBride et al., 1992). While FrzCD homologs from myxobacteria possess highly conserved MCP signaling domains, modest sequence variability about the N-termini seem to correlate with taxonomic structure of myxobacterial genera (Supplementary Figures S8, S9) (McBride et al., 1992; Bustamante et al., 2004). Interestingly, deletion of the N-terminal region of FrzCD *M. xanthus* was found to only minimally impact swarming (Bustamante et al., 2004). From these preliminary observations, we suggest that further investigation on the effect of MeJA on the *frz* signaling pathway of *A.* sp. will provide insight into this unique response and any contributions of the *frz* signaling pathway in the potential plant recruitment of micropredators.

We were also excited to see increased transcription of a putative lytic CHAP amidase during MeJA exposure conditions. Combined with previous induced changes in metabolism and motility, we consider increased production of a lytic enzyme to round out the support for the predatory response of *A.* sp. when exposed to MeJA. Of the other genes upregulated when exposed to MeJA, both the xylan/chitin deacetylase (12-fold increase) and the bifunctional hydroxymethyl pyrimidine kinase (63-fold increase) also stand out due to their previously known association with phytohormone responses (Goyer, 2010; Nagae et al., 2016a, b; Buhian and Bensmihen, 2018). The plant pathogen *Rhizobium radiobacter* demonstrated a 4.6-fold increase in functional xylanase production when exposed to 250 μM MeJA (Jung et al., 2014). To date, cellulose degradation has been exclusively demonstrated by myxobacteria from the suborder Sorangiineae, and not observed from myxobacterium within the suborder Cystobacterineae, which includes *A.* sp. (Sharma et al., 2016; Adaikpoh et al., 2017; Sharma and Subramanian, 2017; Mohr et al., 2018). While increased expression of a xylan degrading enzyme might simply be an opportunistic switch to consume plant cellulose when exposed to MeJA, high homology to chitin deacetylase, NodB, and its role in the biosynthesis of lipo-chitinoligosaccharides (LCOs), or synonymously Nod-factors, suggests a more nuanced impact on symbiosis, as LCOs are chemical entities known to facilitate plant–microbe symbiosis (Buhian and Bensmihen, 2018). Fungal pathogen-induced enrichment of carbohydrate-active enzymes within root microbiomes by members of the genera *Chitinophaga* and *Flavobacterium* has also been associated with disease suppression (Carrion et al., 2019). The only prior association between myxobacteria and LCO biosynthesis, the identification of a homolog for the LCO biosynthetic enzyme NodC from the myxobacterium *Stigmatella aurantiaca*, does not provide additional insight as *A.* sp. does not possess a NodC homolog (Silakowski et al., 1996). Chitin deacetylation has also been associated with accumulation of *N*-glucosamines and a stimulated increase in plant systemic resistance (De Tender et al., 2019).

Also associated with nodule growth and root nodule symbiosis, thiamine biosynthesis is critical to both plant and prokaryote growth (Nagae et al., 2016a, b). Interestingly, MeJA exposure significantly increased expression of a bifunctional hydroxymethyl pyrimidine kinase ThiD homolog involved in two, sequential phosphorylations of 4-amino-5-hydroxymethyl-2-methylpyrimidine (HMP) to generate 4-amino-5-hydroxymethyl-2-methylpyrimidine pyrophosphate (HMP-PP) and decreased transcription of an identified ThiE homolog (threefold, Supplementary Material) responsible for coupling HMP-PP with 4-methyl-5-(β-hydroxyethyl) thiazole (HET-P) to afford thiamine monophosphate (THP) (Begley et al., 1999; Leonardi and Roach, 2004). This change in thiamine-associated features suggests that *A.* sp. accumulates HMP-PP when exposed to MeJA. Interestingly, root nodule symbiosis induces expression of the HET-P biosynthetic gene *THI1* and slightly downregulates expression of *thiD* and *thiE* homologs in the plant *Lotus japonicus* (Zgadzaj et al., 2016). Combined these results provide further evidence implying thiamine biosynthesis as a component of plant–microbe symbiosis and suggest accumulation of HMP-PP by beneficial bacteria and HET-P by plants might benefit precursor pools required for thiamine biosynthesis within the rhizobiome (Nagae et al., 2016a, b). Also of note, a bacterial riboswitch that employs an HMP-PP-binding aptamer has been recently identified and implicated in bacterial regulation of thiamine biosynthesis (Atilho et al., 2019). The regulatory impact of HMP-PP accumulation and its potential to modulate bacterial riboswitches could also contribute to the broad transcriptional impact of MeJA exposure. Provided these results, we are enthusiastic to investigate the symbiotic potential of predatory myxobacteria within the rhizobiome and the roles of LCO and thiamine metabolites.

While the majority of genes downregulated during MeJA exposure were hypothetical, significant repression of genes associated with membrane features and signaling such as a chain-length determining protein responsible for exopolysaccharide biosynthesis, a cyclopropane fatty-acyl-phospholipid synthase, a GntR family transcriptional regulator, and a histidine kinase-like ATPase suggest the broad scope of phytohormone-impacted features (Table 2 and Figure 4). Downregulated specialized metabolite features that might be associated with observed MeJA-induced metabolic shifts included a thiazole/oxazole modified microcin (TOMM) kinase/cyclase fusion protein potentially involved in the biosynthesis of bacteriocin-like metabolites, a SAM-dependent methyltransferase, and a cytochrome p450 (Melby et al., 2011).

Our data demonstrate that myxobacteria demonstrate varying metabolic responses to MeJA exposure and are capable of metabolic modification of MeJA. Interestingly, only one of the four investigated myxobacteria satisfied all three of our suggested criteria supporting plant recruitment of micropredators via the phytohormone MeJA. We suspect subsequent investigations that also include better studied myxobacteria such as the model myxobacterium *M. xanthus* as well as additional signaling metabolites associated with biotic stress responses from plants will provide more insight into associated myxobacterial responses as well as the physiological systems involved. Detection of a potential fatty acid metabolite known to induce myxobacterial motility exclusively within our MeJA exposed samples and MeJA-impacted motility of *A.* sp. provides the first metabolic insight into plant recruitment and maintenance of beneficial micropredators within the rhizobiome. Overall, the combination of impacted motility, metabolism, and lytic enzyme production from our MeJA exposure experiments demonstrate a significant response from the predatory myxobacterium *A.* sp. strain Cb G35. Considering the ≥ fourfold change in transcription for 56 genes and significant impact on metabolism, we conclude that the plant phytohormone MeJA might contribute to recruitment and maintenance of other myxobacteria within rhizobiomes and suggest that the impact of plant exudates on microbial community structure and maintenance requires further investigation.

## Materials and Methods

### Medias and Strains

For all assays, *A.* sp. strain Cb G35 (DSM 52696), *C. coralloides* strain M2 (DSM 2259), *C. ferrugineus* strain Cb fe23 (DSM 52764), and *N. pusilla* strain Na p20 (DSM 53165) initially obtained from the German Collection of Microorganisms (DSMZ) in Braunschweig were used. Myxobacteria were grown on VY/2 agar (5 g/L baker’s yeast, 1.36 g/L CaCl_2_, 0.5 mg/L vitamin B_12_, 15 g/L agar, pH 7.2).

### MeJA Exposure Experiments

For MeJA exposure conditions, required volumes of filter sterilized, (±)-MeJA (Cayman Chemical) from a 150 mM stock prepared in DMSO were added to autoclaved medium at 55°C. For RNA-seq and LC-MS/MS analysis, *A.* sp. was cultivated on VY/2 media supplemented with 10 μM MeJA where appropriate and grown at 30°C for 7 days. While similar MeJA exposure experiments conducted with the plant pathogen *R. radiobacter* utilized MeJA concentrations as high as 250 μM, a more conservative MeJA concentration of 10 μM informed by literature investigating plant responses to exogenous MeJA exposure as well as stress induced production of jasmonates was utilized throughout (Mueller et al., 1993; Blechert et al., 1995; Vijayan et al., 1998; Jung et al., 2014).

### Metabolite Extraction and Analysis

After 5–7 days of cultivation, myxobacterial plates were manually diced and extracted with excess EtOAc. Pooled EtOAc was filtered and dried *in vacuo* to provide crude extracts for LC-MS/MS analysis. LC-MS/MS analysis of the extracted samples was performed on an Orbitrap Fusion instrument (Thermo Scientific, San Jose, CA, United States) controlled with Xcalibur version 2.0.7 and coupled to a Dionex Ultimate 3000 nanoUHPLC system. Samples were loaded onto a PepMap 100 C18 column (0.3 mm × 150 mm, 2 μm, Thermo Fisher Scientific). Separation of the samples was performed using mobile phase A (0.1% formic acid in water) and mobile phase B (0.1% formic acid in acetonitrile) at a rate of 6 μL/min. The samples were eluted with a gradient consisting of 5–60% solvent B over 15 min, ramped to 95% B over 2 min, held for 3 min, and then returned to 5% B over 3 min and held for 8 min. All data were acquired in positive ion mode. Collision-induced dissociation (CID) was used to fragment molecules, with an isolation width of 3 *m/z* units. The spray voltage was set to 3600 V, and the temperature of the heated capillary was set to 300°C. In CID mode, full MS scans were acquired from *m/z* 150 to 1200 followed by eight subsequent MS2 scans on the top eight most abundant peaks. The orbitrap resolution for both the MS1 and MS2 scans was 120,000. The expected mass accuracy was < 3 ppm.

### GNPS, NAP, and XCMS-MRM Analysis

Generated data were converted to. mzXML files using MS-Convert and mass spectrometry molecular networks were generated using the GNPS platform^1^ (Wang et al., 2016). A figure of the corresponding Cytoscape-rendered molecular network is provided as Supplementary Material (Shannon et al., 2003). LC-MS/MS data for this analysis were also deposited in the MassIVE Public GNPS dataset^2^. For subsequent NAP analysis, the cluster index (127) that included 225.148 *m/z* identified as MeJA and confirmed via standard was submitted to the NAP_CCMS2 (version 1.2.5) workflow. Control experiments with MeJA supplemented media and no myxobacteria were run for 7 days, extracted, and analyzed to confirm the absence of any oxidized MeJA analogs; only saponification of MeJA to jasmonic acid was observed after 7 days. Pairwise analysis of converted .mzXML files was done using XCMS-MRM and the default HPLC/Orbitrap (136) parameters. Within the XCMS-MRM result tables, determination of MeJA-impacted detected features was afforded by filtering results for those with a ≥ fivefold change and *p* ≤ 0.05.

### Motility Assays

To monitor swarm expansion rates, the bacteria were cultured on their respective media as described above. After 24 h of incubation, 5 mM stock solutions of MeJA and decanoic acid were spotted onto the solid media, to a concentration of 0.17% v/v, around cells and swarm diameters were recorded daily for 4 days. DMSO was used as vehicle control for comparison. A minimum of six replicates were included in all motility assays. PRISM v7.0d was used to measure the statistical significance of changes in swarm expansion rates across the strains using two-way ANOVA and the Dunnett’s multiple comparisons test to compare simple effects within rows.

### RNAseq Analysis

Total RNA was isolated from the samples using the RNeasy PowerSoil Total RNA Kit (Qiagen) following the manufacturer’s instructions; 500 mg sample was used for extractions. The concentration of total RNA was determined (Supplementary Table S1) using the Qubit^®^ RNA Assay Kit (Life Technologies). For rRNA depletion, first, 1000 ng of total RNA was used to remove the DNA contamination using Baseline-ZERO^TM^ DNase (Epicentre) following the manufacturer’s instructions followed by purification using the RNA Clean and Concentrator-5 columns (Zymo Research). DNA free RNA samples were used for rRNA removal by using RiboMinus^TM^ rRNA Removal Kit (Bacteria; Thermo Fisher Scientific) and final purification was performed using the RNA Clean and Concentrator-5 columns (Zymo Research). rRNA depleted samples were used for library preparation using the KAPA mRNA HyperPrep Kits (Roche) by following the manufacturer’s instructions. Following the library preparation, the final concentration of each library (Supplementary Table S1) was measured using the Qubit^®^ dsDNA HS Assay Kit (Life Technologies), and average library size for each was determined using the Agilent 2100 Bioanalyzer (Agilent Technologies). The libraries were then pooled in equimolar ratios of 0.75 nM, and sequenced paired end for 300 cycles using the NovaSeq 6000 system (Illumina). RNAseq analysis was performed using ArrayStar V15. All sequencing services were provided by MR DNA, Molecular Research LP (Shallowater, TX, United States) Raw data fastq files from these experiments are deposited in the Sequence Read Archive at the National Center for Biotechnology Information and are publicly available (PRJNA555342). All *A.* sp. genes with MeJA-impacted transcription ≥ fourfold change at 99% confidence are provided as a Supplementary Material that also provides linear total RPKM values, DNA sequences, and protein IDs for all replicates.

## Data Availability Statement

The datasets generated for this study can be found in the Sequence Read Archive at the National Center for Biotechnology Information PRJNA555342.

## Author Contributions

BA, SA, and HA performed the experiments. SM and JS conducted and advised all mass spectrometry. BA and DS designed the experiments, and wrote the manuscript. DS conceived and supervised the project.

## Conflict of Interest

The authors declare that the research was conducted in the absence of any commercial or financial relationships that could be construed as a potential conflict of interest.

## Footnotes

1 http://gnps.ucsd.edu

2 ftp://massive.ucsd.edu/MSV000083118
