# Supplementary material for: Myxobacterial Response to Methyljasmonate Exposure Indicates Contribution to Plant Recruitment of Micropredators
Source: Front Microbiol. 2020 Jan 28;11:34. doi: 10.3389/fmicb.2020.00034 (PMC6997564; doi:10.3389/fmicb.2020.00034)
Supplement: Supplementary file 1 [file Image_1.PDF]

Myxobacterial Response to Methyljasmonate Exposure Indicates Contribution to Plant Recruitment of Micropredators

Barbara I. Adaikpoh, Shukria Akbar, Hanan Albataineh, Sandeep K. Misra, Joshua S. Sharp, and D. Cole Stevens *

Supplemental Figures

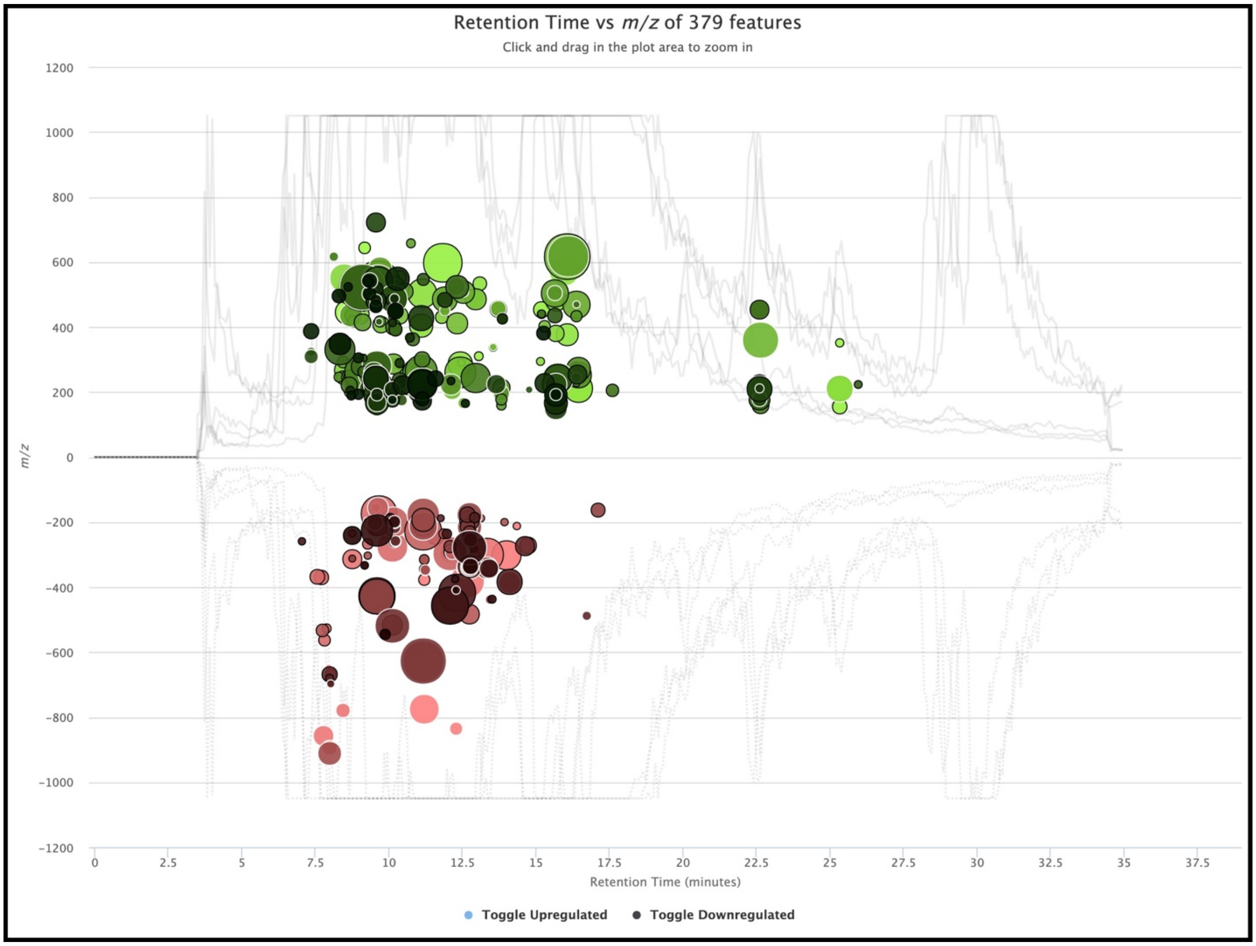

Supplemental Figure 1: Cloud plot rendered by XCMS from LC-MS/MS data collected from extracts of *Archangium* sp. strain Cb G35 comparing MeJA exposed samples to unexposed controls with P-values P≥0.05 and fold change ≥5-fold filtered. Increased features depicted in green with positive m/z values, decreased features depicted in red with negative m/z values.

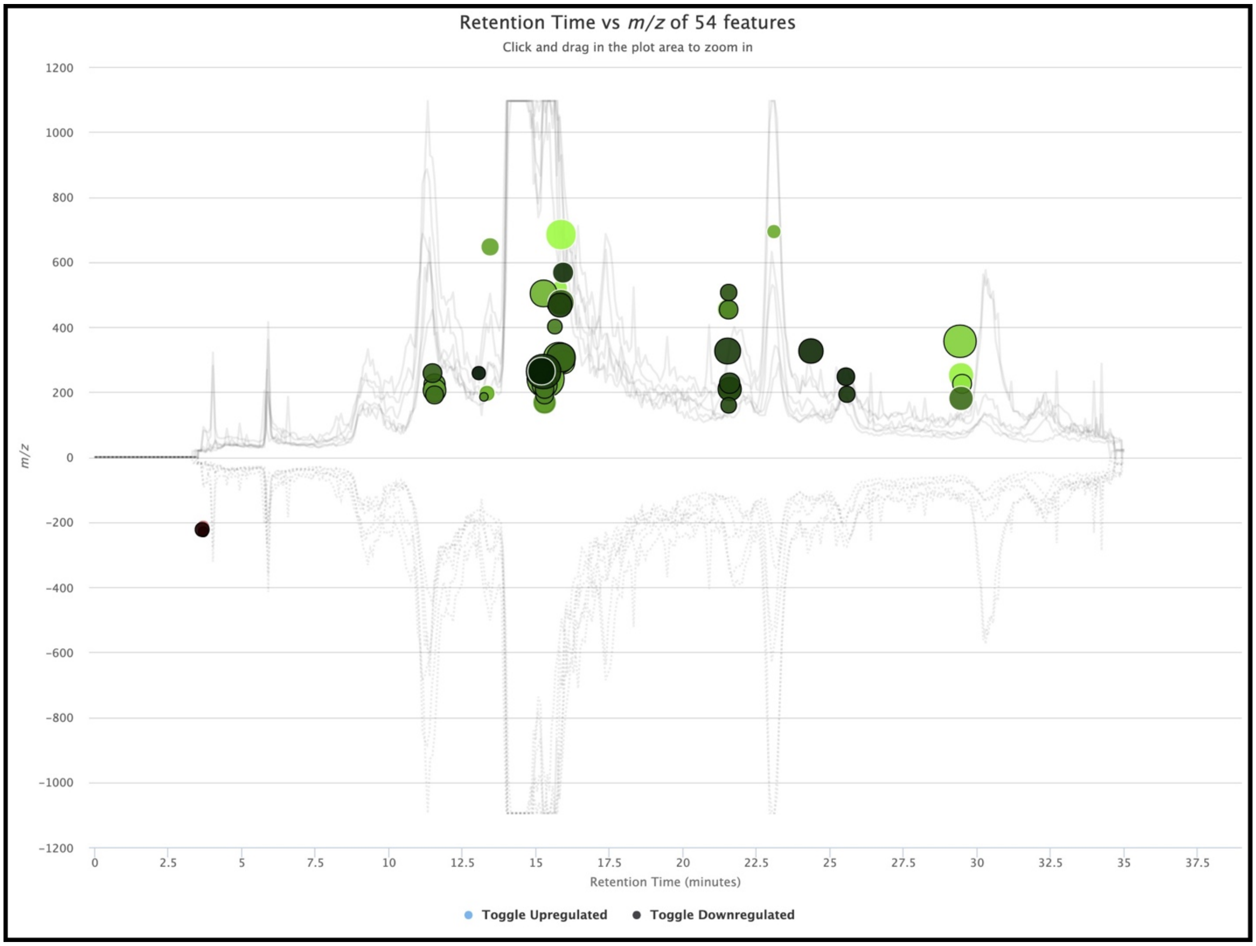

Supplemental Figure 2: Cloud plot rendered by XCMS from LC-MS/MS data collected from extracts of *Corallococcus coralloides* comparing MeJA exposed samples to unexposed controls with P-values P≥0.05 and fold change ≥5-fold filtered. Increased features depicted in green with positive m/z values, decreased features depicted in red with negative m/z values.

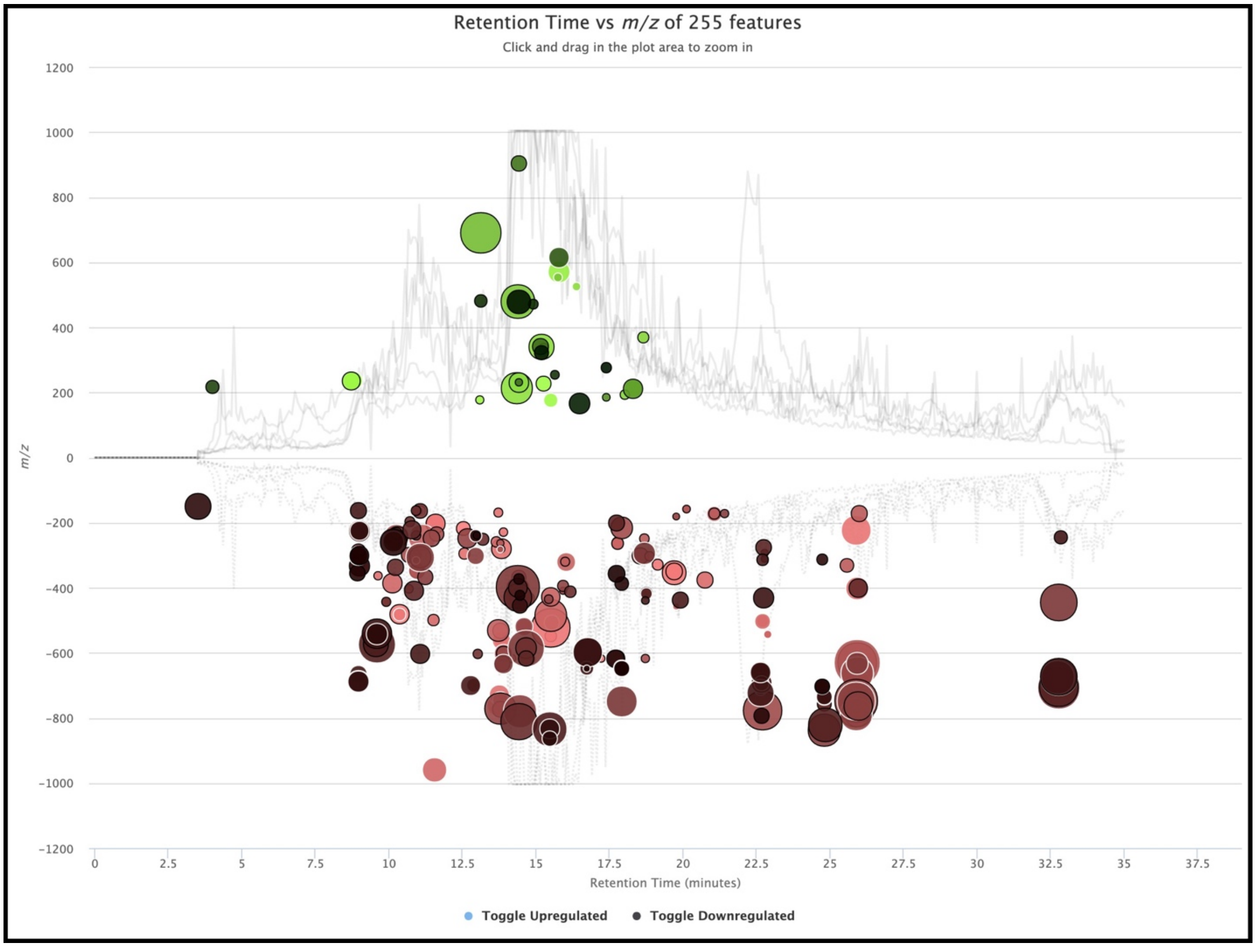

Supplemental Figure 3: Cloud plot rendered by XCMS from LC-MS/MS data collected from extracts of *Nannocystis pusilla* comparing MeJA exposed samples to unexposed controls with P-values P≥0.05 and fold change ≥5-fold filtered. Increased features depicted in green with positive m/z values, decreased features depicted in red with negative m/z values.

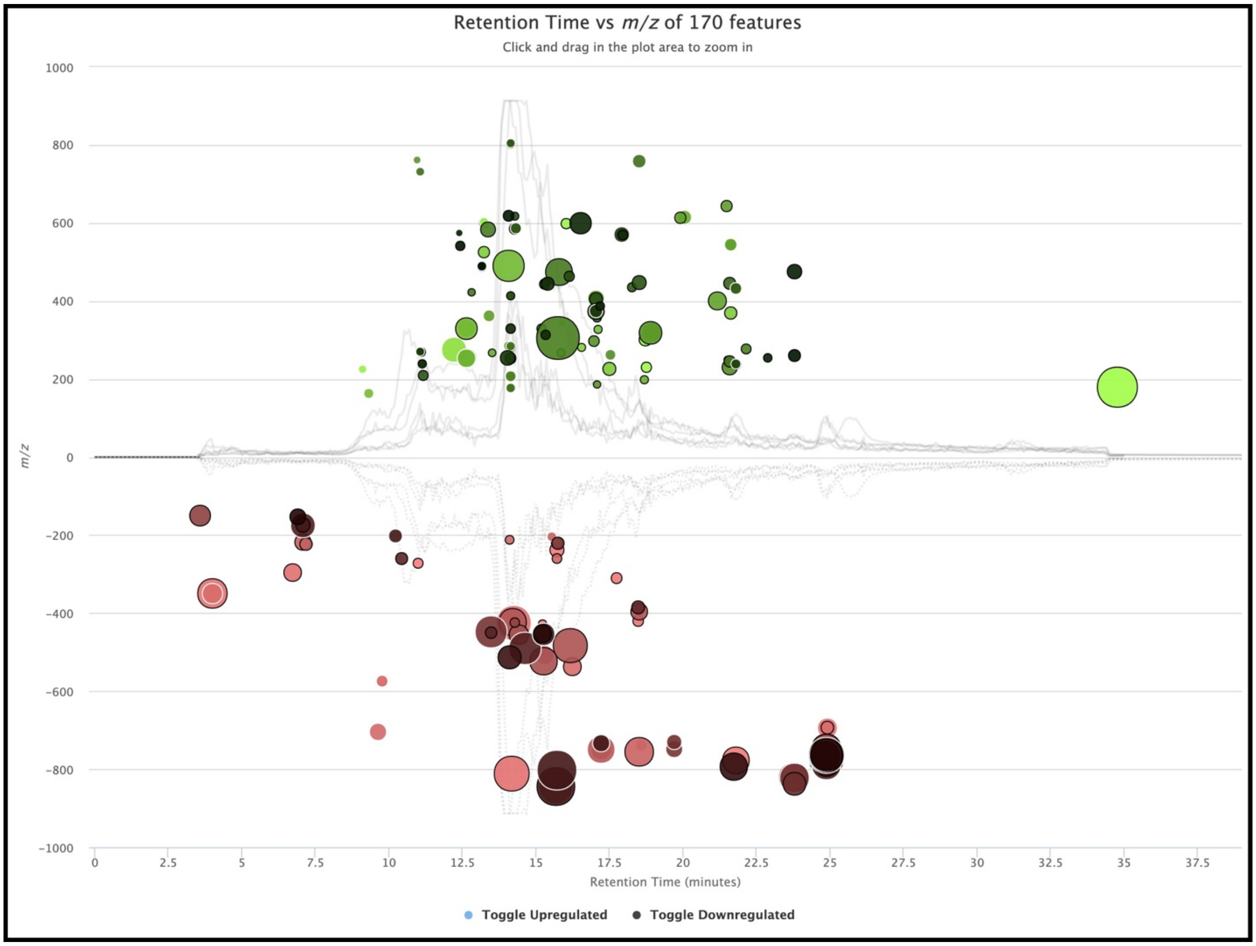

Supplemental Figure 4: Cloud plot rendered by XCMS from LC-MS/MS data collected from extracts of *Cystobacter ferrugineus* comparing MeJA exposed samples to unexposed controls with P-values P≥0.05 and fold change ≥5-fold filtered. Increased features depicted in green with positive m/z values, decreased features depicted in red with negative m/z values.

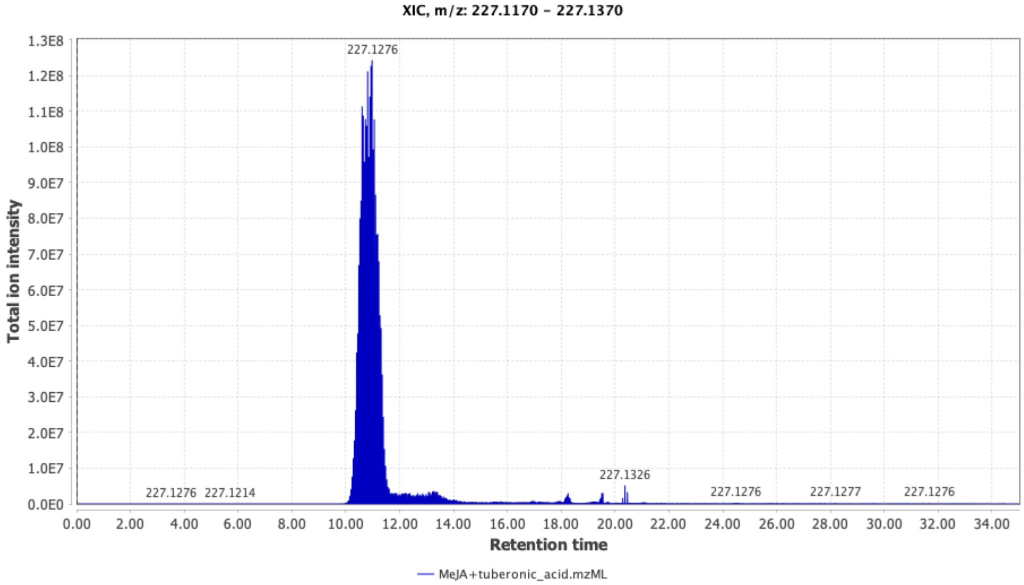

Supplemental Figure 5: Extracted ion chromatograph from 12-hydroxy-jasmonic acid standard LC-MS/MS data (227.1276 m/z).

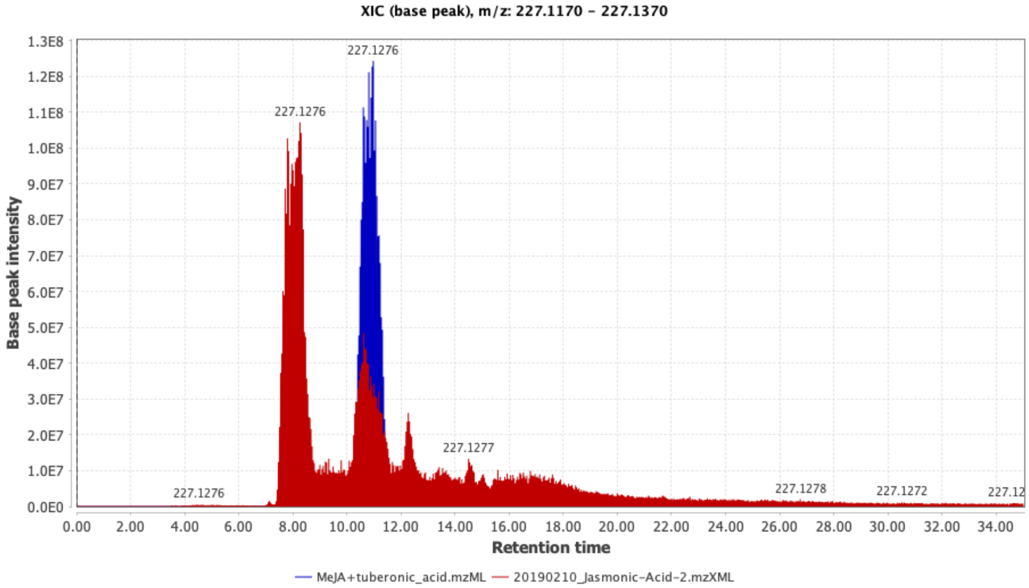

Supplemental Figure 6: Overlay of extracted ion chromatograph from 12-hydroxy-jasmonic acid standard (blue) and *A.* sp. exposed to MeJA extract LC-MS/MS data (227.1276 m/z). While overlapping peaks at retention times around 10min are apparent, fragmentation patterns (SI Figure 7) are not identical confirming the absence of 12-hydroxy-jasmonic acid in *A.* sp. extracts.

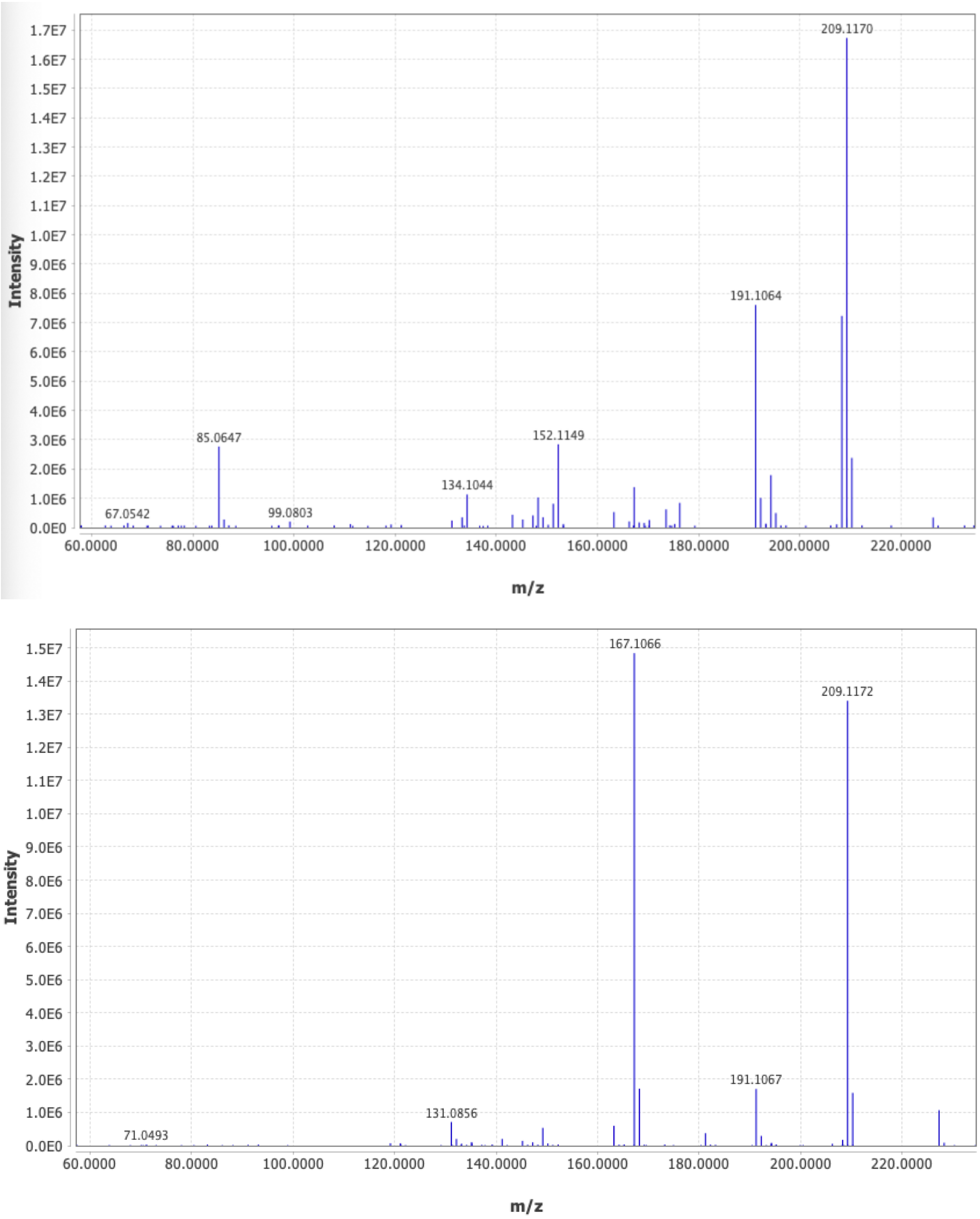

Supplemental Figure 7: MS/MS fragmentation patterns for features with identical retention times (~10min; SI Figure 6) detected at 227.1276 m/z for a 12-hydroxy-jasmonic acid standard (top) and MeJA-exposed *A.* sp. extracts (bottom). Dissimilar fragmentation confirms the absence of 12-hydroxy-jasmonic acid in MeJA-exposed *A.* sp. extracts.

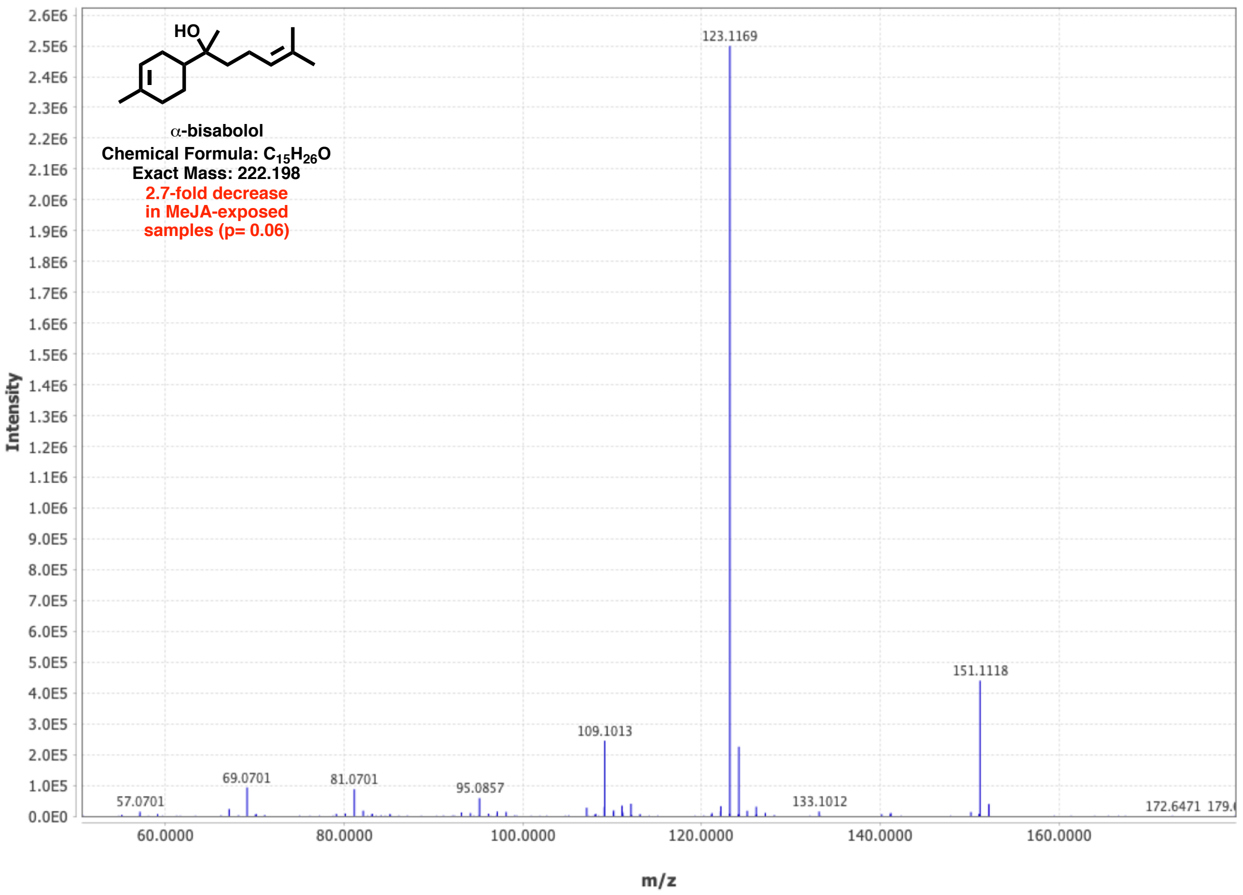

Supplemental Figure 8: MS/MS spectrum for metabolite predicted by GNPS to be α-bisabolol (205.195 m/z; [M+H-H_2O]) rendered in MZmine.

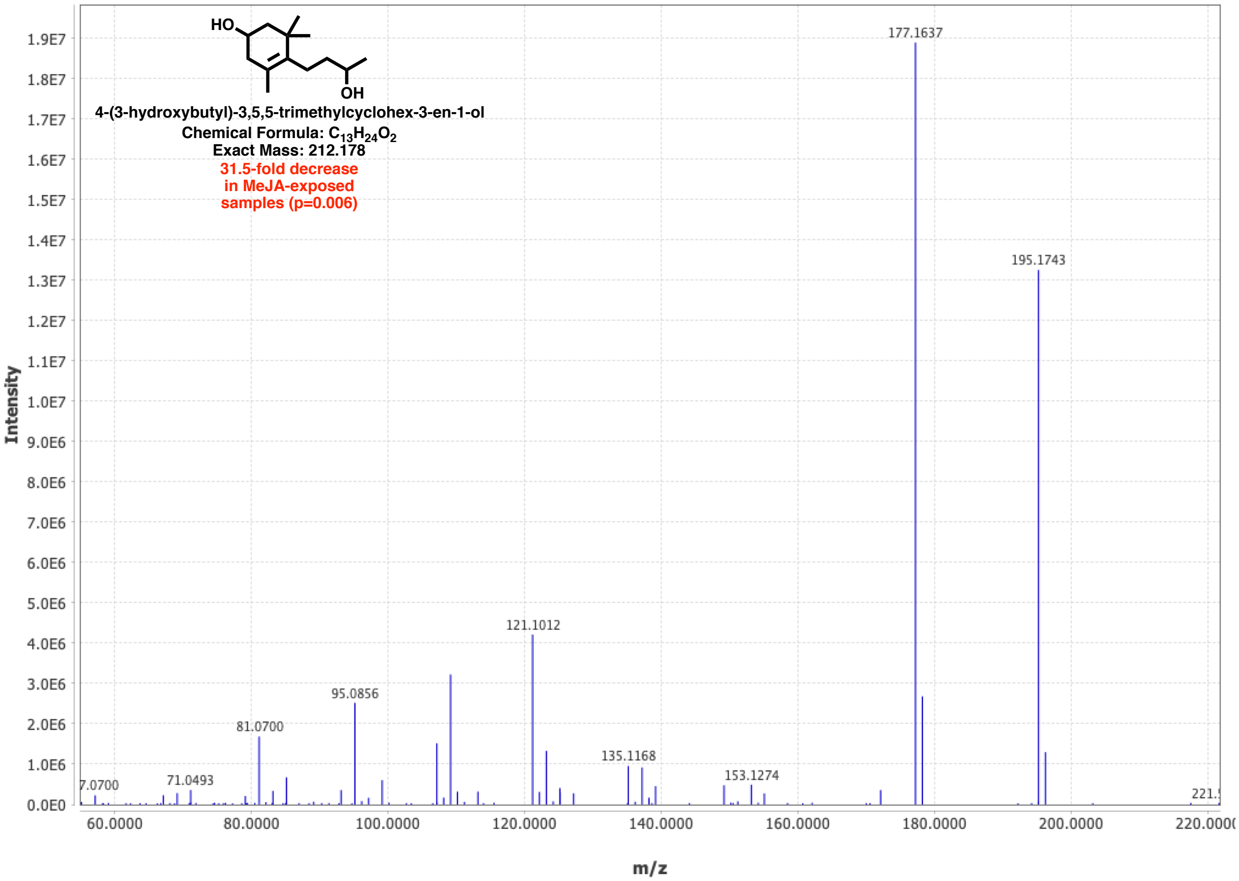

Supplemental Figure 9: MS/MS spectrum for metabolite predicted by GNPS to be 4-(3-hydroxybutyl)-3,5,5-trimethylcyclohex-3-en-1-ol (213.185 m/z; [M+H]) rendered in MZmine.

```
Anaeromyxobacter dehalogenans ref|WP_011419671.1   MRDPSELDKPKKKPARAARPARKAAVPAG------------DALQVEAFLRSFAAGEHGA   48
       Stigmatella aurantiaca ref|WP_013376336.1   ----MSLDTPNEKSVKPRAP-KKAAVAKPP-----AKSNTPALKPLSDTLRSVLAGNLQA   50
           Archangium gephyra ref|WP_047858236.1   ---MSPADTKNEVVPTE-------------GAAAPRSKKAGSLKPLTDTVIAVLAGNLEA   44
        Archangium sp. Cb G35 ref|WP_073562522.1   ---MSPADTKNEVVPTE-------------GAAAPRSKKAGSLKPLTDTVIAVLAGNLEA   44
         Archangium violaceum ref|WP_043395526.1   ---MSPADTKNEVVPTE-------------GAAAPRSKKAGSLKPLTDTVIAVLAGNLEA   44
      Cystobacter ferrugineus ref|WP_071900439.1   ---MSLADSKNDFSATD-------------G--ASRSKKAGSLKPITDTVIAVLAGNLEA   42
           Cystobacter fuscus ref|WP_002632095.1   ---MSLADSKTDFSAAD-------------G--ASRSKKAGSLKPITDTVIAVLAGNLEA   42
    Corallococcus coralloides ref|WP_128797051.1   ----MSLESPNEKPAKSRGA-RKSPGAKAGAESVIANTVTDPFKPFTDTLMSVLAGNLNA   55
           Myxococcus xanthus ref|WP_140792515.1   ----MSLDTPNEKPAGKARA-RKAPASKAGATN--AASTSSSTKAITDTLLTVLSGNLQA   53
                                                          :. ..                                 .   : :. :*:  *

Anaeromyxobacter dehalogenans ref|WP_011419671.1   ARLDVASFADPMLRGIAEAANAAADHAVEQLDTSRHRVQVVTAGVDEAIEAMIRLVIQGD   108
       Stigmatella aurantiaca ref|WP_013376336.1   RITPEIVTGELA--DV----AILLNQVLERFSDSEHRKQVAAQEIDQALDSLIALVREGD   104
           Archangium gephyra ref|WP_047858236.1   RVPKNALEDDAA--DL----AQLFNQLLDRHASSERRKQVAAQEIDQAIDALITLVREGD   98
        Archangium sp. Cb G35 ref|WP_073562522.1   RVPKNAVDEDAA--DL----AQLFNQLLDRHASSERRKQVAAQEIDQAIDALITLVREGD   98
         Archangium violaceum ref|WP_043395526.1   RVPKNAVDDDAA--DL----AQLFNQLLDRHASSERRKQVAAQEIDQAIDALITLVREGD   98
      Cystobacter ferrugineus ref|WP_071900439.1   RVHKNALDEDAA--DL----ANLFNQLLERFAVSEHRKQVAAQEIDQAVDALIALGRDGD   96
           Cystobacter fuscus ref|WP_002632095.1   RVHKNALDEDAA--DL----ANLFNQLLERFAVSEHRKQVAAQEIDQAVDALISLGRDGD   96
    Corallococcus coralloides ref|WP_128797051.1   RVPQDRVQADTT--GF----GHLLNQVLEQFSSAEHRKQVAAQEIDQALDSLIILVREGD   109
           Myxococcus xanthus ref|WP_140792515.1   RVPKELVGESGA--EL----AHLLNQVLDQFSASEHRKHVAAQEIDQALDALIGLVREGD   107
                                                            .     .        :: :::   :.:* :*.:  :*:*::::* *  :**

Anaeromyxobacter dehalogenans ref|WP_011419671.1   LSGTLRLGVSDAALTPLIAGIGQVMETLKRFVTEIREAALQLSTSSAEVLAAATQNESST   168
       Stigmatella aurantiaca ref|WP_013376336.1   LS-RWTTTTEDPQLAPLLEGFGKVIETLRTFVREINEAALRLSSSANQVLAASTQHETSS   163
           Archangium gephyra ref|WP_047858236.1   LA-QWNTSTEDAQLGPLLEGFGKVIETLRTFVREINEAALRLSSSANQVLAASTQHETSS   157
        Archangium sp. Cb G35 ref|WP_073562522.1   LA-QWNTSTEDAQLGPLLEGFGKVIETLRTFVREINEAALRLSSSANQVLAASTQHETSS   157
         Archangium violaceum ref|WP_043395526.1   LA-QWNTSTEDAQLGPLLEGFGKVIETLRTFVREINEAALRLSSSANQVLAASTQHETSS   157
      Cystobacter ferrugineus ref|WP_071900439.1   LA-RWNTTTEDAQLAPLLEGFGKVIETLRTFVREINEAALRLSSSSNQVLSASTQHETSS   155
           Cystobacter fuscus ref|WP_002632095.1   LA-RWNTTTEDAQLAPLLEGFGKVIETLRTFVREINEAALRLSSSSNQVLSASTQHETSS   155
    Corallococcus coralloides ref|WP_128797051.1   LS-RWNTSTEDPQLGPLLEGFGKVIETLRIFVREINEAALRLSSSANQVLAASTQHETSS   168
           Myxococcus xanthus ref|WP_140792515.1   LS-RWNTTTEDPQLGPLLEGFGKVIETLRTFVREINEAALRLSSSANQVLAASTQHETSS   166
                                                   *:      ..*  * **: *:*:*:***: ** **.****:**:*: :**:*:**:*:*:

Anaeromyxobacter dehalogenans ref|WP_011419671.1   SAQASAIHETTATMEELKGASHQIAENAQMVAAIAEQTLTGAKQGEGAIKSFMSSMEKVR   228
       Stigmatella aurantiaca ref|WP_013376336.1   TEQAAAIHETTATMEELKHASAQIAENAGAVARVAEETLGAARAGRGAIAEFIQAMQQIR   223
           Archangium gephyra ref|WP_047858236.1   TEQAAAIHETTATMEELKHASAQIAENAGSVARVAEETLGAARAGRGAIGEFIQAMQQIR   217
        Archangium sp. Cb G35 ref|WP_073562522.1   TEQAAAIHETTATMEELKHASAQIAENAGSVARVAEETLGAARAGRGAIGEFIQAMQQIR   217
         Archangium violaceum ref|WP_043395526.1   TEQAAAIHETTATMEELKHASAQIAENAGSVARVAEETLGAARAGRGAIGEFIQAMQQIR   217
      Cystobacter ferrugineus ref|WP_071900439.1   TEQAAAIHETTATMEELKHASAQIAENAGSVARVAEETLGAARAGRGAIGEFIQAMQQIR   215
           Cystobacter fuscus ref|WP_002632095.1   TEQAAAIHETTATMEELKHASAQIAENAGSVARVAEETLGAARAGRGAIGEFIQAMQQIR   215
    Corallococcus coralloides ref|WP_128797051.1   TEQAAAIHETTATMEELKHASAQIAENAGSVARVAEETLGAARAGRGAIGEFIQAMQQIR   228
           Myxococcus xanthus ref|WP_140792515.1   TEQAAAIHETTATMEELKHASAQIAENAGSVARVAEETLGAARAGRGAIGEFIQAMQQIR   226
                                                   : **:************* ** ******  ** :**:** .*: *.*** .*:.:*:::*

Anaeromyxobacter dehalogenans ref|WP_011419671.1   HNAVEVDDAIAKLSKRVERIGTVVEVIDEIADRSDLLALNAALEGAKAGEAGRGFSIVAA   288
       Stigmatella aurantiaca ref|WP_013376336.1   SDGIAVADAITKLSKRVERIGTVVEVIDEIADRSDLLALNAALEGSRAGEAGKGFSIVAA   283
           Archangium gephyra ref|WP_047858236.1   SDGVAVADSITKLSKRVERIGTVVEVIDEIADRSDLLALNAALEGSRAGEAGKGFSIVAA   277
        Archangium sp. Cb G35 ref|WP_073562522.1   SDGVAVADSITKLSKRVERIGTVVEVIDEIADRSDLLALNAALEGSRAGEAGKGFSIVAA   277
         Archangium violaceum ref|WP_043395526.1   SDGVAVADSITKLSKRVERIGTVVEVIDEIADRSDLLALNAALEGSRAGEAGKGFSIVAA   277
      Cystobacter ferrugineus ref|WP_071900439.1   SDGVAVADSIAKLSKRVERIGTVVEVIDEIADRSDLLALNAALEGSRAGEAGKGFSIVAA   275
           Cystobacter fuscus ref|WP_002632095.1   SDGVAVADSITKLSKRVERIGTVVEVIDEIADRSDLLALNAALEGSRAGEAGKGFSIVAA   275
    Corallococcus coralloides ref|WP_128797051.1   SDGVAVADSISKLSKRVERIGTVVEVIDEIADRSDLLALNAALEGSRAGEAGKGFSIVAA   288
           Myxococcus xanthus ref|WP_140792515.1   SDGVAVADSIAKLSKRVERIGTVVEVIDEIADRSDLLALNAALEGSRAGEAGKGFSIVAA   286
                                                    :.: * *:*:**********************************::*****:*******

Anaeromyxobacter dehalogenans ref|WP_011419671.1   EMRRLAENVLESTKEIKNLITEIREATHAAKEASDGNKRMAAEGEQLGGNAMTSVSGILS   348
       Stigmatella aurantiaca ref|WP_013376336.1   EMRRLAENVLDSTKEIKSLITEIREATAAAGGAADASKVATESGEKLGSVAATAVEGILA   343
           Archangium gephyra ref|WP_047858236.1   EMRRLAENVMESTKEIKNLITEIREATAAAGSAADASKEATESGEKLGAVAAQAVEGILA   337
        Archangium sp. Cb G35 ref|WP_073562522.1   EMRRLAENVMESTKEIKNLITEIREATAAAGSAADASKEATESGEKLGAVAAQAVEGILA   337
         Archangium violaceum ref|WP_043395526.1   EMRRLAENVMESTKEIKNLITEIREATAAAGSAADASKEATESGEKLGAVAAQAVEGILA   337
      Cystobacter ferrugineus ref|WP_071900439.1   EMRRLAENVMESTKEIKNLITEIREATAAAGTAAEASKEATESGEKLGAVAAQAVEGILA   335
           Cystobacter fuscus ref|WP_002632095.1   EMRRLAENVMESTKEIKNLITEIREATAAAGAAAEASKEATESGEKLGAVAAQAVEGILA   335
    Corallococcus coralloides ref|WP_128797051.1   EMRRLAENVLESTKEIKNLITEIREATQAAAGAADASKHATESGEKLGAVAAQAVEGILA   348
           Myxococcus xanthus ref|WP_140792515.1   EMRRLAENVLDSTKEIKNLITEIREATAAAAGAAEASKSATESGEKLGAVAAQAVEGILA   346
                                                   *********::******.********* **  *::..*  : .**:**. *  :*.***:

Anaeromyxobacter dehalogenans ref|WP_011419671.1   GIQETSDAARVIHLATQQQRTATEQVVQSMSEIEEVTRQAQTGSKQATGAASELTKLAER   408
       Stigmatella aurantiaca ref|WP_013376336.1   GVQETSDAARVINLATQQQRTATEQVVASMAEIEDVTRQTTQASKQATGASAELTQLASR   403
           Archangium gephyra ref|WP_047858236.1   GVQETSDAARVINLATQQQRTATEQVVASMAEIEDVTRQTTQASKQATGAAAELTQLAGR   397
        Archangium sp. Cb G35 ref|WP_073562522.1   GVQETSDAARVINLATQQQRTATEQVVASMAEIEDVTRQTTQASKQATGAAAELTQLAGR   397
         Archangium violaceum ref|WP_043395526.1   GVQETSDAARVINLATQQQRTATEQVVASMAEIEDVTRQTTQASKQATGAAAELTQLAGR   397
      Cystobacter ferrugineus ref|WP_071900439.1   GVQETSDAARVINLATQQQRTATEQVVASMAEIEDVTRQTTSASKQATGAAAELNQLAGR   395
           Cystobacter fuscus ref|WP_002632095.1   GVQETSDAARVINLATQQQRTATEQVVASMAEIEDVTRQTTSASKQATGAAAELNQLAGR   395
    Corallococcus coralloides ref|WP_128797051.1   GVQETSDAARVINLATQQQRTATEQVVASMAEIEDVTRQTTQASKQATGAAAELTQLASR   408
           Myxococcus xanthus ref|WP_140792515.1   GVQETSDAARVINLATQQQRTATEQVVASMAEIEDVTRQTTQASKQATGAAAELTQLAGR   406
                                                   *:**********:************** **:***:****:  .*******::**.:** *

Anaeromyxobacter dehalogenans ref|WP_011419671.1   LAELVKRFKVE      419
       Stigmatella aurantiaca ref|WP_013376336.1   LAELIKRFKAD      414
           Archangium gephyra ref|WP_047858236.1   LAELIKRFKAD      408
        Archangium sp. Cb G35 ref|WP_073562522.1   LAELIKRFKAD      408
         Archangium violaceum ref|WP_043395526.1   LAELIKRFKAD      408
      Cystobacter ferrugineus ref|WP_071900439.1   LAELIKRFKAD      406
           Cystobacter fuscus ref|WP_002632095.1   LAELIKRFKAD      406
    Corallococcus coralloides ref|WP_128797051.1   LAELIKRFKAD      419
           Myxococcus xanthus ref|WP_140792515.1   LAELIKRFKAD      417
                                                   ****:****.:
```

Supplemental Figure 10: Alignment of FrzCD homologs from myxobacteria rendered by ClustalOmega (v1.2.4).

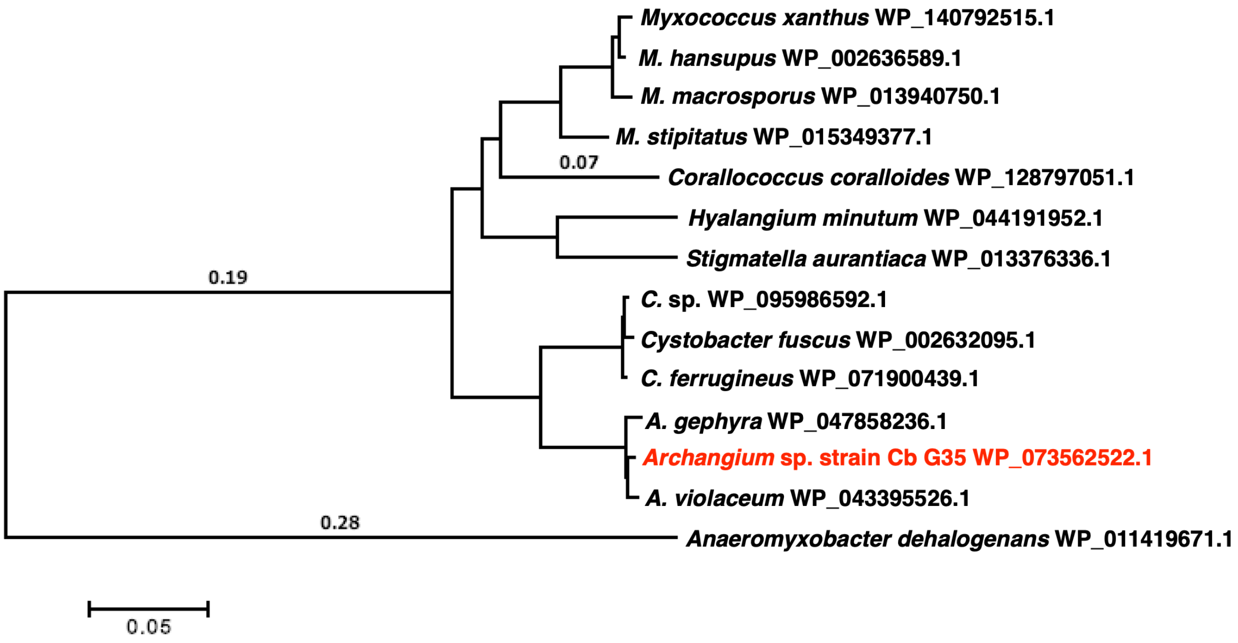

Supplemental Figure 11: Neighbor-joining tree of FrzCD homologues rendered in MEGA7 from aligned sequences in Supplemental Figure 10. Branch lengths <0.05 not depicted.
